# Arbuscular mycorrhizal fungus changes alfalfa response to pathogen infection activated by pea aphid infestation

**DOI:** 10.3389/fmicb.2022.1074592

**Published:** 2023-02-08

**Authors:** Yajie Wang, Yingde Li, Tingyu Duan

**Affiliations:** ^1^State Key Laboratory of Herbage Improvement and Grassland Agro-Ecosystems, Lanzhou University, Lanzhou, China; ^2^Key Laboratory of Grassland Livestock Industry Innovation, Ministry of Agriculture and Rural Affairs, Lanzhou, China; ^3^College of Pastoral Agriculture Science and Technology, Lanzhou University, Lanzhou, China

**Keywords:** AM fungus, plant–pathogen–insect interaction, *Medicago sativa*, *Phoma medicaginis*, pea aphid

## Abstract

**Introduction:**

Arbuscular mycorrhizal (AM) fungi are important for the resistance of plants to insect infestation and diseases. However, the effect of AM fungal colonization of plants response to pathogen infection activated by pea aphid infestation is unknown. Pea aphid (*Acyrthosiphon pisum*) and the fungal pathogen *Phoma medicaginis* severely limit alfalfa production worldwide.

**Methods:**

This study established an alfalfa (*Medicago sativa*)–AM fungus (*Rhizophagus intraradices*)–pea aphid–*P*. *medicaginis* experimental system to clarify the effects of an AM fungus on the host plant response to insect infestation and subsequent fungal pathogen infection.

**Results:**

Pea aphid increased the disease incidence of *P. medicaginis* by 24.94%. The AM fungus decreased the disease index by 22.37% and enhanced alfalfa growth by increasing the uptake of total nitrogen and total phosphorus. The aphid induced polyphenol oxidase activity of alfalfa, and the AM fungus enhanced plant-defense enzyme activity against aphid infestation and subsequent *P. medicaginis* infection. In addition, the AM fungus increased the contents of jasmonic acid and abscisic acid in plants exposed to aphid infestation or pathogen infection. Abscisic acid and genes associated with the gene ontology term “hormone binding” were upregulated in aphid-infested or pathogen-infected alfalfa.

**Discussion:**

The results demonstrate that an AM fungus enhances plant defense and signaling components induced by aphid infestation, which may contribute to improved defense against subsequent pathogen infection.

## 1. Introduction

The interactions of plants with pathogens and herbivorous insects are among the most extensive ecological relationships (Raffa et al., 2020). The incidence of plant disease is often accompanied by insect infestation. Moreover, insects often spread plant diseases by acting as vectors for pathogens (Roumagnac et al., 2015; Davoodi et al., 2018). For some pathogens, such as viruses and fungi, dispersal is largely dependent on the interactions among pathogens, hosts, and vectors (Hohn, 2007; Sugio et al., 2014). Insects can increase, decrease, or have no effect on pathogen infection of plants (Rostás and Hilker, 2002; Simon and Hilker, 2003; Eyles et al., 2007). Thus, plant pathogens and herbivorous insects can cause synergistic effects on the host plant (Sugio et al., 2011).

Arbuscular mycorrhizal (AM) fungi are important components of agroecosystems with important roles in plant resistance to biotic stress, such as that caused by insects or diseases, and abiotic stress tolerance (Babikova et al., 2014; Dreher et al., 2019). In addition, insects can affect AM fungi under host plant mediation in the AM fungi–plant–insect system. Insect feeding reduces plant biomass, plant photosynthesis, and photosynthate transport to the roots. Thus, it affects the acquisition of lipids by AM fungi from the host plant, limiting the colonization of host plants by AM fungi (He et al., 2017; Charters et al., 2020).

In the AM fungus–plant–pathogen interaction system, the AM fungus and pathogen obtain lipids from the host plant to maintain their survival (Jiang et al., 2017). Therefore, pathogens and mycorrhizal fungi potentially compete for host nutrition, parasitic sites, and immunity signal receptors (Zhang et al., 2021). The AM fungi reduce the rate of pathogen infection, thus reducing or delaying the harm to plants (Filion et al., 1999). In addition, AM fungi induce plant defense resistance responses, such as plant hormone signaling and antioxidant enzyme activities (Liu et al., 2018), and regulate the contents of jasmonic acid (JA) and phenylpropanoids in the host (Kloppholz et al., 2011). Moreover, AM fungi upregulate pathogenesis-related (PR) proteins, transcription factors, secondary metabolism, and genes associated with the salicylic acid (SA) pathway (Zhang and Franken, 2014; Gutjahr et al., 2015).

Aphids are major agricultural pests that typically reproduce parthenogenetically or viviparously to rapidly produce large offspring populations (Jaouannet et al., 2015). Pea aphid (*Acyrthosiphon pisum*) causes severe global economic damage to pulse crops (Elbakidze et al., 2010) by feeding directly on phloem sap, damaging the phloem, and is a vector of several harmful viruses (Ng and Perry, 2004; Guo et al., 2012). Pea aphid infests leguminous crops from multiple genera, such as broad bean (*Vicia faba*), lupin (*Lupinus albus*), alfalfa (*Medicago sativa*), and pea (*Pisum sativum*) (Aznar-Fernández et al., 2019; Smith et al., 2021).

Alfalfa is an important forage crop with a high protein content (Mbarki et al., 2018) and comprises the largest area of legume forage cultivated worldwide (Sprent et al., 2017). However, the fungal pathogen *Phoma medicaginis* severely damages alfalfa by causing spring black stem and leaf spot disease. The disease has been reported in the United States (Akamatsu et al., 2008), Canada (Ginns, 1986; Hilton, 2000), India (Alaka and Rao, 1998), Italy (Balmas et al., 2005), Tunisia (Djebali, 2013), and China (Fan et al., 2018). Alfalfa spring black stem and leaf spot disease causes dry matter loss, reduced seed production, decreased forage quality, and poor winter survival (Rhodes and Myers, 1986; Castell-Miller et al., 2008; Sathoff et al., 2020).

Aphids and spring black stem and leaf spot disease usually simultaneously attack alfalfa, causing severe damage (Stout et al., 2006). Few studies have focused on the plant responses to pea aphid infestation and *P. medicaginis* infection, particularly the influence of AM fungi on the plant response to aphid infestation and subsequent pathogen infection. However, the plant response is important for management of aphids and leaf spot disease in alfalfa. A previous study has shown that the AM fungus *Rhizophagus intraradices* promotes alfalfa growth during pea aphid infestation by enhancing plant peroxidase (POD) activity, SA content, and the expression of resistance-related genes (Li et al., 2019). In addition, *R. intraradices* reduces the severity of disease caused by *P. medicaginis* by inducing defense pathways, increasing the JA content, and stimulating the expression of *P. medicaginis* resistance-related genes (Li Y et al., 2021).

In the present study, a greenhouse experiment was established to determine the effect of AM fungus *R. intraradices* on the alfalfa response to the combination infection of pea aphid and *P. medicaginis*. We hypothesized that (1) plant defense profiles will differ with AM fungal colonization, aphid infestation and pathogen infection, and (2) AM fungi will enhance pea aphid-induced plant defense response against subsequent pathogen infection.

## 2. Materials and methods

### 2.1. Plant growth and AM fungus inoculation

Seeds of alfalfa “Longdong,” a popular cultivar grown in China, were obtained from the Forage Seed Testing Centre, Lanzhou, Ministry of Agriculture, and the Rural Ministry of China. The AM fungus *R. intraradices* was purchased from the Bank of Glomeromycota in China (Beijing, China). The inoculum consisted of dry soil that contained AM fungal spores (>100 spores per gram) and mycelia, and white clover (*Trifolium subterraneum*) root fragments. The AM fungus was grown on white clover in a greenhouse (Li Y et al., 2021).

The alfalfa growth medium was mixed with pre-sieved sand and soil (1:3, w/w) and sieved through a 2 mm mesh. The sand and soil were purchased from a flower market in Lanzhou, China. The growth medium components were sterilized by autoclaving at 121°C for 1 h twice in 3 days (Li Y et al., 2021).

**Figure 1:**
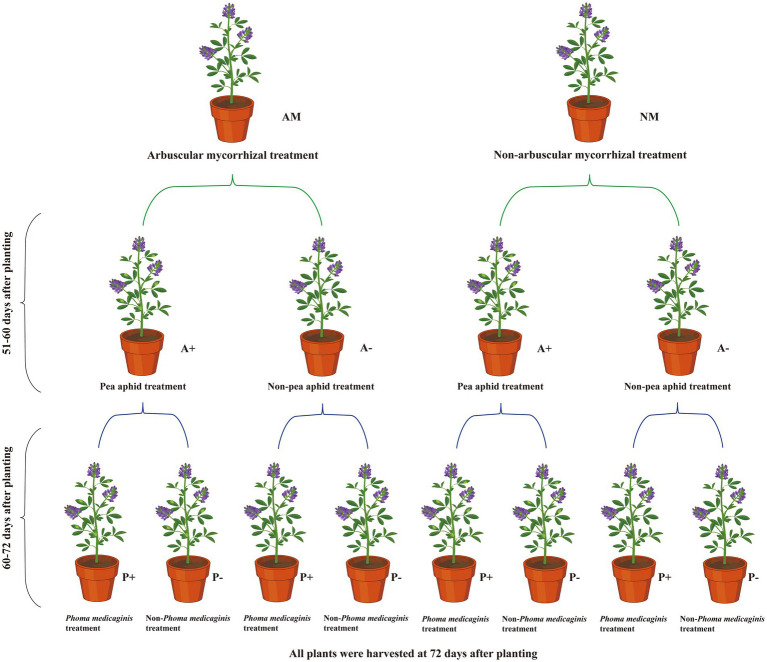
Flow chart showing the experimental design of the study.

A three-stage experiment to examine alfalfa, AM fungus, aphid, and pathogen interactions was established in a greenhouse (Figure 1). Alfalfa seeds were surface sterilized with 10% hydrogen peroxide for 10 min and rinsed three times with reverse osmosis water. The sterilized seeds were placed on wet filter paper and incubated in the dark at 25°C for 48 h. Five germinated seeds were planted in growth medium inoculated with *R. intraradices* (AM) or non-inoculated medium (NM) and thinned to four seedlings after 1 week. Before transplanting the seedlings, inoculum (50 g) from the AM fungus pot cultures was added to the growth medium at a depth of 2–3 cm in 16 pots (arbuscular mycorrhizal treatment, AM). For the non-AM control treatment (non-mycorrhizal, NM), 50 g of the growth medium was added to growth medium in 16 pots (12 cm height × 10 cm diameter at the top × 8 cm diameter at the base; Gao et al., 2018).

### 2.2. Insect and disease treatment

Pea aphid individuals were captured at an alfalfa field on the Yuzhong campus of Lanzhou University (Lanzhou, China). A single adult aphid was transferred to broad bean plants for culture, and the aphid offspring were selected for morphological and molecular identification (Zhang et al., 2018). The pathogen *P. medicaginis* was isolated from field-grown diseased alfalfa plants, and identified by morphological and molecular characterization (Gao et al., 2018). The pathogen was grown on potato dextrose agar and conidia were harvested to generate an inoculum of 6 × 10^6^ conidia/mL (Gao et al., 2018).

At 51 days after planting (dap), alfalfa plants grown in AM- or NM-inoculated medium were infested with five similarly sized pea aphid adults (A+) or not infested (A−). At 60 dap, half of the mycorrhizal and aphid-infested alfalfa plants were sprayed with 20 ml of *P. medicaginis* inoculum (6 × 10^6^ conidia/mL; P+). The remaining half were sprayed with 20 ml sterilized water (P−). The plants were covered with black plastic bags for 48 h to maintain high levels of moisture on the plants to facilitate pathogen infection. After removal of the black plastic bags, adult pea aphids were selected to replenish the alfalfa plants with up to five adults.

There were eight treatments with four pots each as replicates for a total of 32 pots. The plants were harvested 12 days after inoculation with the pathogen. Before harvest, all leaves of each pot were observed to assess disease incidence and calculate the disease index for leaf spot caused by *P. medicaginis*. Disease severity was estimated using a five-class visual scale based on the percentage of the leaf surface covered by pustules as follows: 0, no symptom of infection; 1, 0.1–5% of the leaf area covered with leaf spots; 2, >5–20%; 3, >20–50%; 4, >50–75%; and 5, >75%. The disease index was calculated using the following equation: $\mathrm{DI}=100\times \frac{\sum_{n=i}^{i}\left(n\times Ln\right)}{Ln\times 5}$ , where *i* is the disease severity scale (0 to 5), Ln is the number of leaves with each disease severity, and *n* is the total number of leaves (Li Y et al., 2021).

The experiment was conducted in a glasshouse with irradiance in the range of 200–900 μmol m^−2^ s^−1^ during the growth period. The average temperatures were 22–29°C (day) and 14–25 (night).

### 2.3. Plant harvest and measurement of samples

At harvest, 0.1 g fresh shoots from each pot were randomly sampled for total RNA extraction, flash-frozen immediately in liquid nitrogen, and stored at −80°C. In addition, 0.5 g fresh shoots from each pot were sampled for measurement of enzyme activities, comprising POD, catalase (CAT), and polyphenol oxidase (PPO), as previously described (Gao et al., 2018). Approximately 0.4 g fresh shoots from each pot were sampled to measure the contents of JA, SA, nitric oxide (NO), trypsin inhibitor, and abscisic acid (ABA) using ELISA kits (mlbio; Shanghai Enzyme-linked Biotechnology, Shanghai, China; Li Y et al., 2021). Fresh shoots (0.5 g) were used to reisolate the pathogen. The remaining fresh shoot tissues were used to measure the dry weight. Thus, the total plant-shoot dry weight was calculated from the fresh and dry weight ratio. Subsequently, 0.2 g dry shoots were digested in 5 ml H_2_SO_4_. The nitrogen and phosphorus contents in shoots were determined using a flow injection analyzer (FIAstar 5,000 Analyzer; FOSS, Höganäs, Sweden). As previously described, approximately 0.15 g fresh roots from each AM treatment pot were used for AM colonization tests (Supplementary Figure S1). Roots were cut into 1-cm-long segments and cleared in 10% KOH for 40 min at 60°C. The samples were treated with 1 M HCl for 30 s, washed three times with distilled water, and stained in trypan blue overnight at room temperature. The stained roots were washed three times with distilled water and maintained in a solution of 5% lactic acid:glycerin:water (1:1:1, v/v/v). The root samples were observed to determine the degree of mycorrhizal colonization with a compound microscope (SOFTOP BH200M-R, Zhejiang, China; Phillips and Hayman, 1970; Giovannetti and Mosse, 1980).

### 2.4. Transcriptome analysis of plant leaves

Total RNA was isolated from leaf tissues of three biological replicates per treatment (NMA−P−, NMA+P−, NMA−P+, NMA+P+, AMA−P−, AMA+P−, AMA−P+, and AMA+P+) using TRIzol® (Thermo Fisher Scientific, Waltham, MA, United States) following the manufacturer’s instructions. Residual genomic DNA was removed from the RNA samples using DNase I (Takara Bio, Kusatsu-Shiga, Japan). The RNA quality and quantity were determined using a 2,100 Bioanalyzer (Agilent Technologies, Santa Clara, CA, USA) and a NanoDrop ND-2000 spectrophotometer (Thermo Fisher Scientific). Only high-quality RNA samples (OD_260/280_ = 1.8–2.2, OD_260/230_ ≥ 2.0, RIN ≥ 6.5, 28S:18S ≥ 1.0, >2 μg) were used to construct the RNA-sequencing (RNA-Seq) libraries.

Accordingly, 1 μg total RNA was used to create RNA-Seq transcriptome libraries using the TruSeq™ RNA Sample Preparation Kit (Illumina, San Diego, CA, USA) following the manufacturer’s instructions. Total mRNA was isolated by the polyA selection method using oligo(dT) beads and fragmented using fragmentation buffer. The SuperScript™ Double-Stranded cDNA Synthesis Kit (Invitrogen, Carlsbad, CA, USA) was used to synthesize double-stranded cDNA with random hexamer primers (Illumina). The synthesized cDNA was end-repaired, phosphorylated, and “A” base enriched following the manufacturer’s instructions. After cDNA quantification, 200–300 bp target fragments were amplified by PCR using Phusion DNA polymerase (New England Biolabs, Ipswich, MA, USA) for 15 PCR cycles. The libraries were paired-end sequenced with an Illumina HiSeq X/NovaSeq 6,000 sequencer (2 × 150 bp read length).

The raw paired-end reads were trimmed by SeqPrep^1^ and subjected to quality control with Sickle^2^ using the default parameters. The clean reads were mapped to the *M. sativa* reference genome^3^ using TopHat (http://tophat.cbcb.umd.edu/, version 2.0.0) software (Trapnell and Pachter, 2009). The generated raw sequence dataset was submitted to the National Center for Biotechnology Information Short Read Archive (SRA) database under accession number PRJNA859548.

### 2.5. Differential expression analysis and functional enrichment

The RSEM software package^4^ was used to normalize the gene expression level as fragments per kilobase of exon per million mapped reads (FPKM). Applying the P_adjust_ < 0.05 and |log_2_ fold change| ≥ 2 criteria (O'Connell et al., 2012), analyses of differentially expressed genes (DEGs) between two samples (NMA − P− vs. AMA−P−, NMA−P− vs. NMA+P−, NMA−P− vs. NMA+P−, NMA−P− vs. NMA+P+, AMA−P− vs. AMA+P−, AMA−P− vs. AMA+P−, AMA−P− vs. AMA+P+, NMA+P− vs. AMA+P−, NMA−P+ vs. AMA−P+, and NMA+P+ vs. AMA+P+) were performed using the “DESeq2” R package with raw counts in the R statistical environment (Zhuang et al., 2018; Supplementary Table S1). Gene ontology enrichment analysis was conducted with Fisher exact tests (*Padjust* < 0.05). The DEGs with Bonferroni-corrected *p*-value ≤ 0.05 were considered to be significantly enriched in the functional-enrichment analysis of gene ontology (GO) terms and Kyoto Encyclopedia of Genes and Genomes (KEGG) pathways. Goatools^5^ and KOBAS (http://kobas.cbi.pku.edu.cn/home.do; Xie et al., 2011) were used to determine GO and KEGG enrichment.

### 2.6. Real-time quantitative reverse-transcription PCR validation

Ten genes were randomly selected for measurement by real-time quantitative reverse-transcription PCR (qRT-PCR) with three independent biological replicates for each treatment. The cDNA was synthesized from the total RNA used for RNA-Seq using the FastKing gDNA Dispelling RT SuperMix (TianGen, Beijing, China) following the manufacturer’s instructions. The primers were designed with Beacon Designer 7.9 and are listed in Supplementary Table S2. The qRT-PCR reactions used SuperReal PreMix Plus (SYBR Green; TianGen).

### 2.7. Statistical analysis

Data for AM colonization, shoot fresh and dry weights, and contents of JA, SA, NO, ABA, and trypsin inhibitor are presented as the mean ± standard error of the mean (SEM) of four pots (i.e., four biological replicates). The shoot total nitrogen and shoot total phosphorus contents, disease incidence, disease index, and the activities of CAT, POD, and PPO are presented as the mean ± SEM of three biological replicates. All data were subjected to analysis of variance (ANOVA) using R statistical program (version 4.2.0). Comparison between the individual means was conducted with Tukey’s honestly significant difference (HSD) test (*p* < 0.05). The percentage AM colonization was arcsine-transformed to achieve normality. The RNA-Seq data were analyzed using the free online platform Majorbio Cloud.^6^

## 3. Results

### 3.1. AM fungal colonization, plant growth, and nitrogen and phosphorus uptake

The percentage AM fungal colonization ranged from 41.67 to 46.67% of the inoculated alfalfa plants, whereas the non-inoculated plants had no observable mycorrhizal structures in their roots. Pea aphid and *P. medicaginis* did not significantly affect percentage AM fungal colonization (Table 1; Supplementary Table S3). Moreover, AM fungal colonization increased the fresh and dry shoot weights of alfalfa by 76.34 and 31.73%, respectively. The pathogen *P. medicaginis* decreased the fresh shoot weight of non-mycorrhizal and mycorrhizal alfalfa by 42.73 and 58.32%, respectively (Table 1; Supplementary Table S3).

**Table 1:** AM colonization, shoot fresh weight, shoot dry weight, shoot total N and shoot total P of *Medicago sativa* inoculated with *Rhizophagus intraradices* (AM), infested with *Acyrthosiphon pisum* (A+) and infected with *Phoma medicaginis* (P+) or un-inoculated with *R. intraradices* (NM), un-infested with *A. pisum* (A−) and un-infected with *P. medicaginis* (P−).

Treatments	Mycorrhizal colonization (%)	Shoot fresh weight (g)	Shoot dry weight (g)	Shoot total N (mg·pot^−1^)	Shoot total P (mg·pot^−1^)
NMA−P−	0.00 ± 0.00	4.22 ± 0.58 b	0.69 ± 0.07 b	23.28 ± 3.67 b	3.04 ± 1.12 b
NMA+P−	0.00 ± 0.00	4.62 ± 0.57 b	0.78 ± 0.14 b	28.88 ± 7.35 b	3.28 ± 0.29 b
NMA−P+	0.00 ± 0.00	3.16 ± 0.85 c	0.77 ± 0.16 b	34.31 ± 5.70 b	4.41 ± 1.36 b
NMA+P+	0.00 ± 0.00	1.90 ± 0.30 c	0.78 ± 0.09 b	29.91 ± 4.99 b	2.62 ± 0.46 b
AMA−P−	41.67 ± 1.67 a	7.31 ± 1.31 a	1.09 ± 0.18 a	35.56 ± 1.94 a	3.17 ± 0.19 b
AMA+P−	45.00 ± 1.67 a	8.27 ± 1.49 a	1.23 ± 0.24 a	45.34 ± 2.83 a	6.55 ± 1.43 a
AMA−P+	46.67 ± 2.72 a	3.74 ± 0.88 bc	0.91 ± 0.16 a	32.24 ± 11.19 a	3.36 ± 1.08 b
AMA+P+	46.67 ± 3.85 a	2.75 ± 0.66 bc	0.98 ± 0.13 a	40.17 ± 5.87 a	7.19 ± 1.90 a

Different letters represent significant difference at *p* < 0.05, respectively, as determined by Tukey’s HSD test.

The AM fungal colonization increased the shoot total nitrogen content of alfalfa by 31.74% (*p* = 0.0475). Aphid infestation significantly increased the total phosphorus content of mycorrhizal alfalfa shoots (*p* = 0.0147; Table 1; Supplementary Table S3).

### 3.2. Disease severity

**Figure 2:**
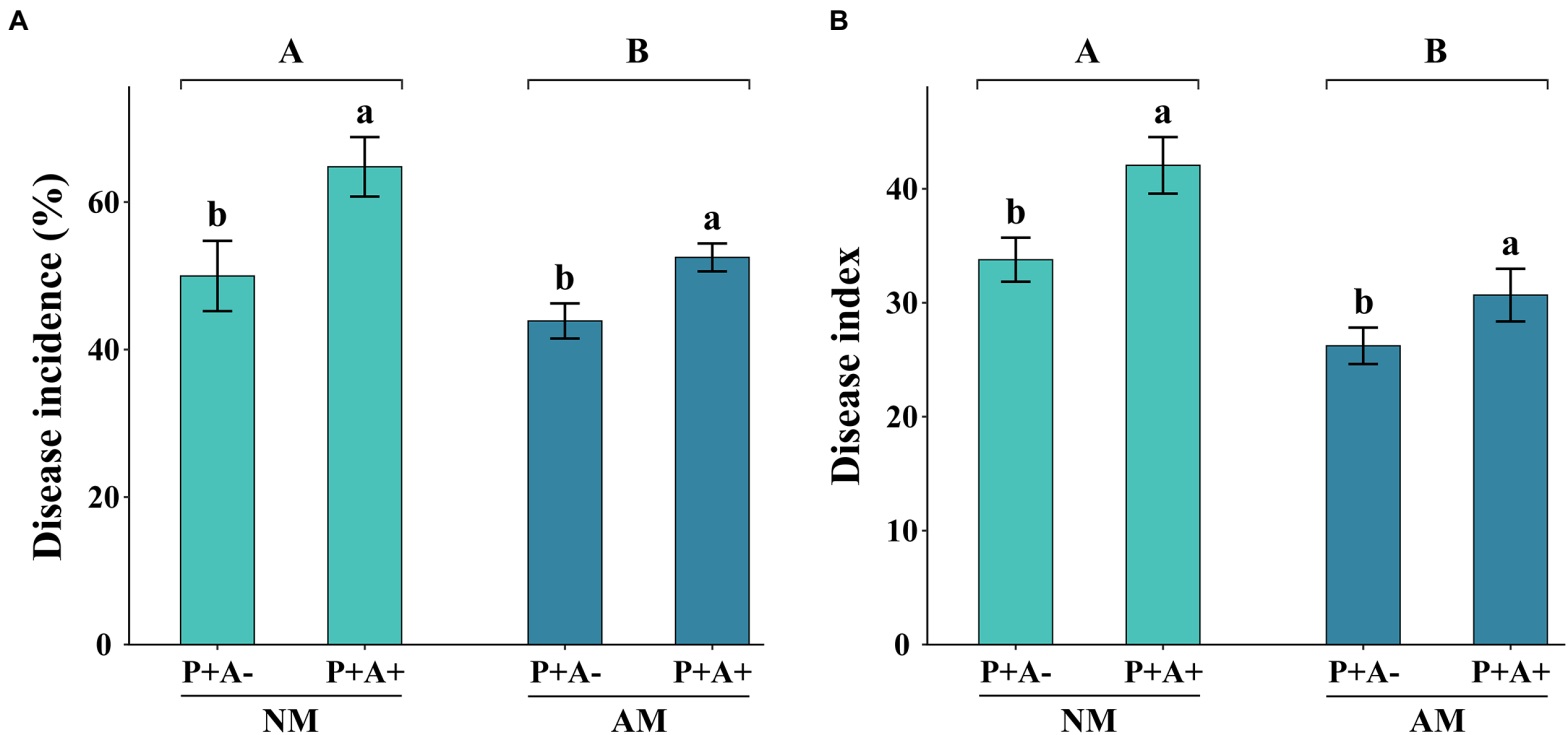
**(A)** Disease incidence and **(B)** disease index of *Medicago sativa* inoculated with *Rhizophagus intraradices* (AM) and infested with *Acyrthosiphon pisum* (A+) or non-inoculated with *R. intraradices* (NM) and non-infested with *A. pisum* (A−). All values are the mean ± SEM of three biological replicates. Different letters above pairs of bars indicate a significant difference in the comparison at *p* < 0.05. Asterisks indicate a significant difference between P + A+ and P + A− as determined by Tukey’s HSD test. SEM, standard error of the mean.

One week after pathogen inoculation, alfalfa infected by *P. medicaginis* showed typical disease symptoms, whereas non-inoculated plants showed no observable symptoms. The disease incidence was 24.94% higher in aphid-infested than non-infested plants (*p =* 0.0097; Figure 2A). The disease incidence of non-mycorrhizal alfalfa was 19.05% higher than that of mycorrhizal alfalfa (*p =* 0.0294; Figure 2A). In addition, the disease index of mycorrhizal plants was 24.97% lower than that of non-mycorrhizal alfalfa (*p* = 0.002; Figure 2B). The disease index of alfalfa was 24.52% higher in aphid-infested alfalfa than non-infested plants (*p* = 0.0166; Figure 2B).

### 3.3. Activities of plant defense enzymes

**Figure 3:**
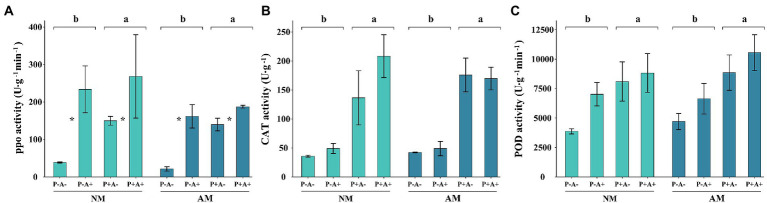
**(A)** PPO activity, **(B)** CAT activity, and **(C)** POD activity of *Medicago sativa* inoculated with *Rhizophagus intraradices* (AM), infected with *Phoma medicaginis* (P+), and infested with *Acyrthosiphon pisum* (A+), or non-inoculated with *R. intraradices* (NM), non-infected with *P. medicaginis* (P−), and non-infested with *A. pisum* (A−). All values are the mean ± SEM of three biological replicates. Different letters above pairs of bars indicate a significant difference in the comparison at *p* < 0.05. In **(A)** asterisks indicate a significant difference between P−A− and P− +, or P+A− and P+A+, as determined by Tukey’s HSD test. SEM, standard error of the mean; PPO, polyphenol oxidase; CAT, catalase; POD, peroxidase.

Aphid infestation increased the activity of PPO by 143.51% compared with that in non-infested alfalfa (*p* = 0.0017). Infection with *P. medicaginis* increased the activity of PPO by 74.13% (*p* = 0.0446; Figure 3A). Similarly, infection with *P. medicaginis* increased the activities of CAT (*p* = 0.0047; Figure 3B) and POD (*p* = 0.042; Figure 3C) in alfalfa by 273.8 and 59.65%, respectively (Supplementary Table S3). AM fungus colonization had no effect on activities of plant defense enzymes.

### 3.4. Contents of SA, JA, ABA, NO, and trypsin inhibitor in alfalfa plants

**Figure 4:**
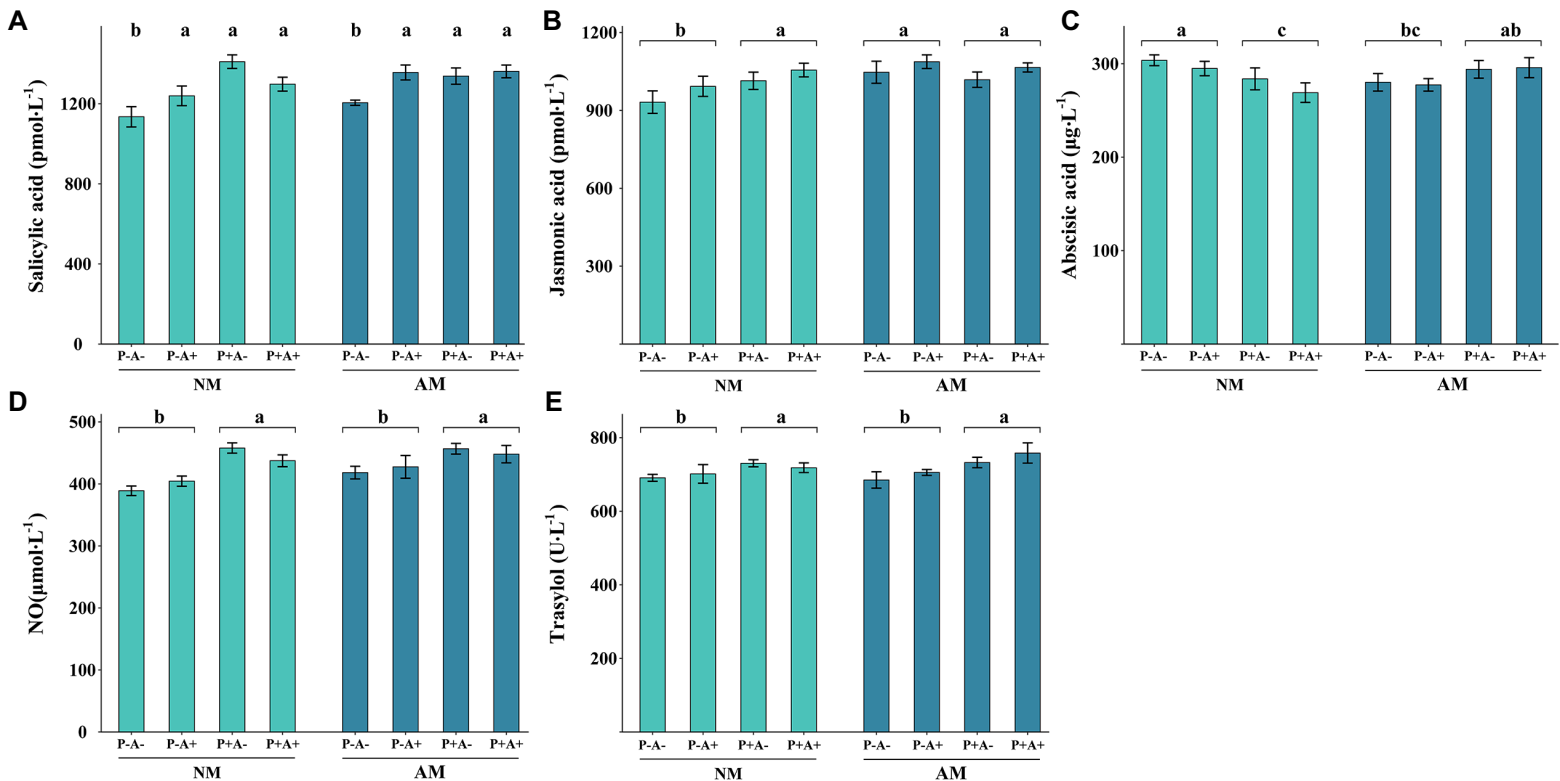
**(A)** SA, **(B)** JA, **(C)** ABA, **(D)** NO, and **(E)** trypsin inhibitor contents of *Medicago sativa* inoculated with *Rhizophagus intraradices* (AM), infected with *Phoma medicaginis* (P+), and infested with *Acyrthosiphon pisum* (A+), or non-inoculated with *R. intraradices* (NM), non-infected with *P. medicaginis* (P−), and non-infested with *A. pisum* (A−). All values are the mean ± SEM of four biological replicates. Different letters above bars **(A)** or pairs of bars **(B–E)** indicate a significant difference in the comparison at *p* < 0.05, as determined by Tukey’s HSD test. SEM, standard error of the mean; SA, salicylic acid; JA, jasmonic acid; ABA, abscisic acid; NO, nitrous oxide.

The SA content was significantly higher in alfalfa infested with pea aphid and infected with *P. medicaginis* than in alfalfa that were not infested with pea aphids or infected with the pathogen (*p* = 0.0038). Colonization by AM fungi did not significantly affect the SA content of alfalfa plants (Figure 4A). Aphid infestation had no effect on JA content, whereas AM colonization alone or in combination with pathogen infection increased the JA content (*p* = 0.0491; Figure 4B). Aphid infestation had no influence on ABA content, whereas AM colonization and pathogen infection together increased the ABA content (*p* = 0.0063; Figure 4C). The NO content (*p* < 0.0001; Figure 4D) and trypsin inhibitor content (*p* = 0.0047; Figure 4E) were only affected by pathogen infection, which increased their levels (Supplementary Table S3).

### 3.5. RNA-Seq and mapping

RNA-sequencing of the leaf transcriptome with an Illumina HiSeq X platform generated 47,309,123 raw reads (maximum 58,300,788, minimum 41,604,716). After removing low-quality and adapter sequences (bases with <20 mass value), 48,677,795, 45,600,933, 49,066,624, 46,863,595, 45,203,664, 44,447,226, 49,016,621, and 45,697,048 average clean reads were obtained from the NMA−P−, NMA+P−, NMA−P+, NMA+P+, AMA−P−, AMA+P−, AMA−P+, and AMA+P+ treatments, respectively. The Q20 and Q30 percentages were >97.88 and >93.82%, respectively. The average GC contents in the eight treatments were similar (approximately 42%). The clean reads were mapped to the *M. sativa* reference genome, resulting in a 43.03–92.65% mapping rate and the low mapping rate samples were pathogen-infected treatments (Supplementary Table S4). A total of 24 samples were obtained 166.4 Gb clean data, the clean data of all samples are reached 6.05 Gb.

### 3.6. Analysis of DEGs

**Figure 5:**
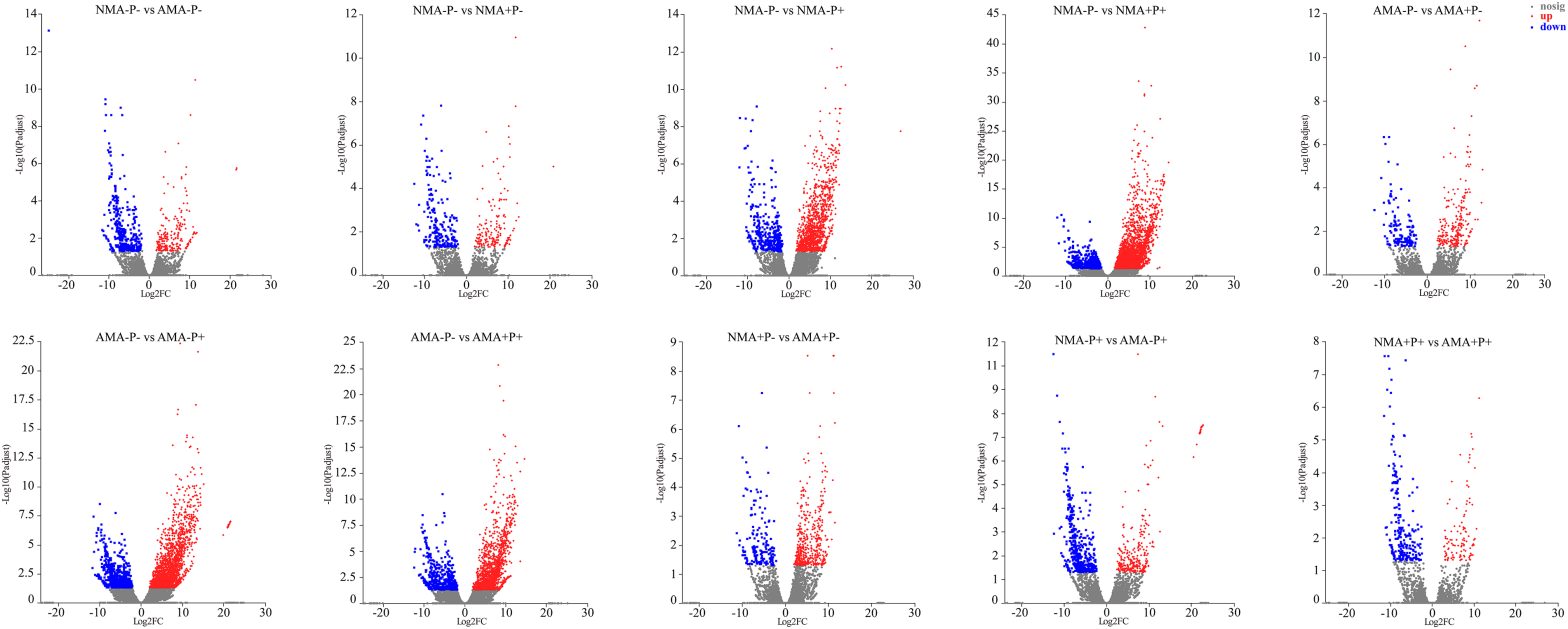
Analysis of DEGs in alfalfa in the comparisons NMA−P− vs. AMA−P−, NMA−P− vs. NMA+P−, NMA−P− vs. NMA−P+, NMA−P− vs. NMA+P+, AMA−P− vs. AMA+P−, AMA−P− vs. AMA−P+, AMA−P− vs. AMA+P+, NMA+P− vs. AMA+P−, NMA−P+ vs. AMA−P+, and NMA+P+ vs. AMA+P+. The *x*-axis represents the gene expression fold changes between any two groups. The *y*-axis represents the statistical test value for the difference in gene expression [−log_10_(*P*_adjust_)]. The scattered points represent each gene. No significant difference is indicated by gray, whereas significant upregulation and downregulation are indicated by red and blue, respectively. DEGs, differentially expressed genes.

**Figure 6:**
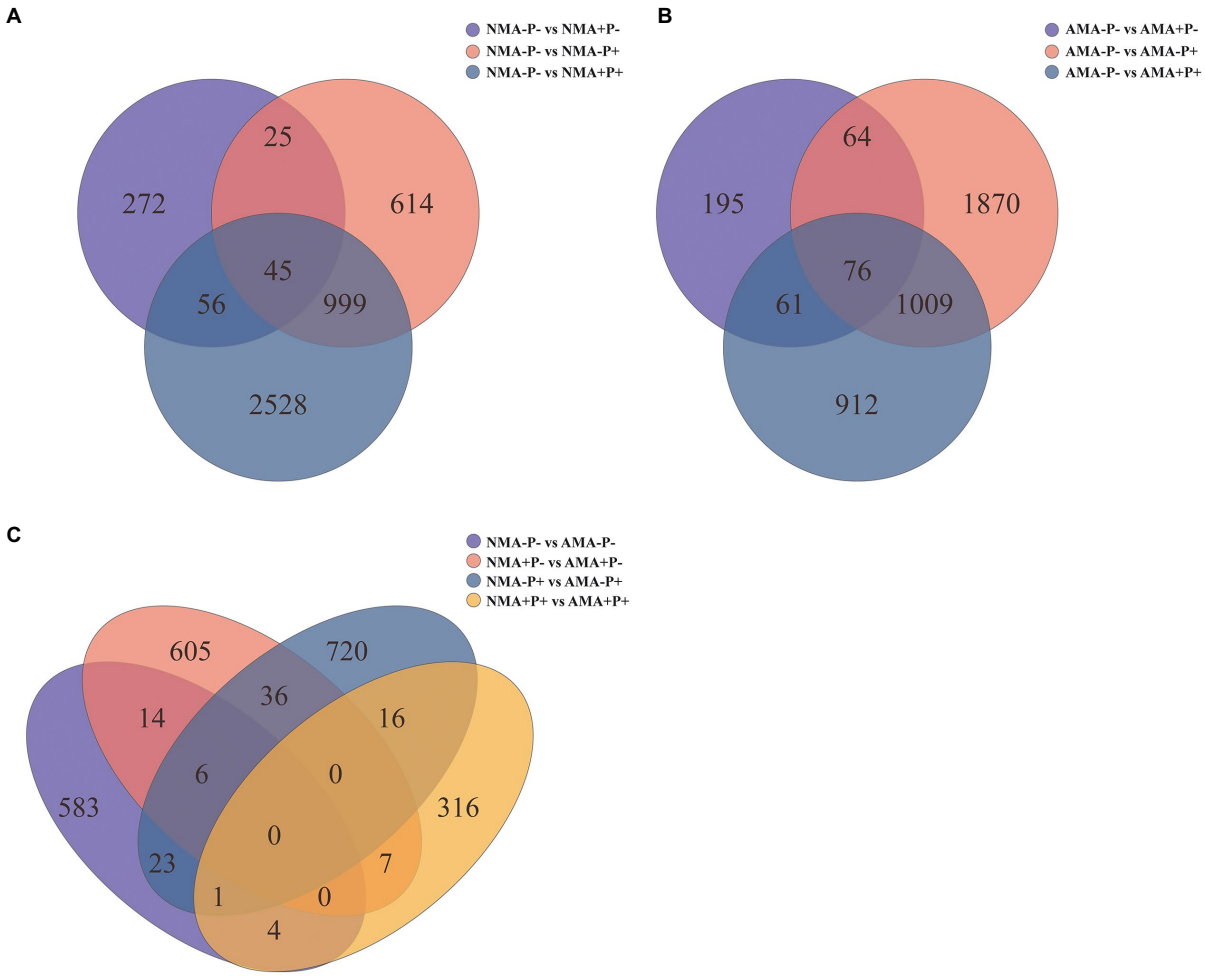
Venn diagram of **(A)** the three gene sets NMA−P− vs. NMA+P−, NMA−P− vs. NMA−P+, and NMA−P− vs. NMA+P+, **(B)** the three gene sets AMA−P− vs. AMA+P−, AMA−P− vs. AMA−P+, and AMA−P− vs. AMA+P+, and **(C)** the four gene sets NMA−P− vs. AMA−P−, NMA+P− vs. AMA+P−, NMA−P+ vs. AMA−P+, and NMA+P+ vs. AMA+P+.

There were 13,627 DEGs were identified across all treatments, of which 344 DEGs to 3,628 DEGs in the comparison between each treatments. Of these DEGs, 234, 486, 309, and 114 were upregulated, whereas 397, 182, 493, and 230 were downregulated, respectively in the comparisons AM fungus, aphid, pathogen and the combination of the three treatments (NMA−P− vs. AMA−P−, NMA+P− vs. AMA+P−, NMA−P+ vs. AMA−P+, and NMA+P+ vs. AMA+P+), respectively (Figure 5; Table 2). A Venn diagram identified 5 to 76 common DEGs in the comparisons between each treatment (Figure 6).

**Table 2:** Counts of differentially expressed genes in alfalfa with AM fungus, aphids and pathogen treatments.

Groups	Control group	Treat group	Differentially Expressed genes number	Up-regulated gene number	Down-regulated gene number
NMA−P− vs. AMA−P−	NMA−P−	AMA−P−	631	234	397
NMA−P− vs. NMA+P−	NMA−P−	NMA+P−	398	176	222
NMA−P− vs. NMA−P+	NMA−P−	NMA−P+	1,683	1,267	416
NMA−P− vs. NMA+P+	NMA−P−	NMA+P+	3,628	2,954	674
AMA−P− vs. AMA+P−	AMA−P−	AMA+P−	396	257	139
AMA−P− vs. AMA-P+	AMA−P−	AMA−P+	3,019	2,217	802
AMA−P− vs. AMA+P+	AMA−P−	AMA+P+	2058	1,545	513
NMA+P− vs. AMA+P−	NMA+P-	AMA+P−	668	486	182
NMA−P+ vs. AMA-P+	NMA−P+	AMA−P+	802	309	493
NMA+P+ vs. AMA+P+	NMA+P+	AMA+P+	344	114	230

### 3.7. Gene ontology enrichment analysis

**Figure 7:**
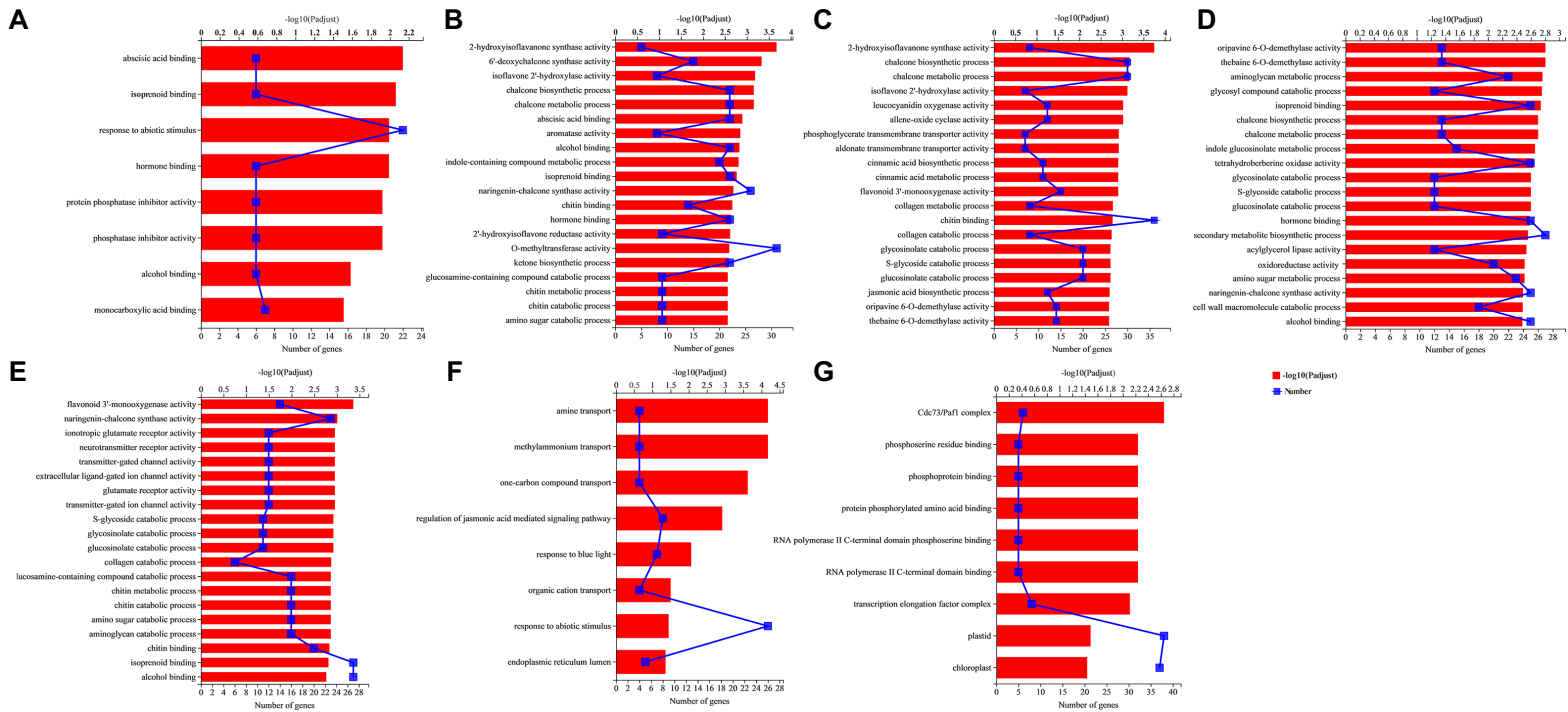
GO enrichment analysis in the comparisons **(A)** NMA−P− vs. NMA+P−, **(B)** NMA−P− vs. NMA−P+, **(C)** NMA−P− vs. NMA+P+, **(D)** AMA−P− vs. AMA−P+, **(E)** AMA−P− vs. AMA+P+, **(F)** NMA+P− vs. AMA+P−, and **(G)** NMA−P+ vs. AMA+P−. The top 20 GO terms are displayed based on differentially expressed genes (*p*_adjust_ < 0.05). The upper *x*-axis shows the significance level of enrichment [−log_10_(*P*_adjust_)]; the larger the value of −log_10_(*P*_adjust_), the greater the enrichment. The lower *x*-axis shows the number of genes. The *y*-axis shows the GO term. GO, gene ontology.

Aphid infestation enriched 8 GO terms in NM treatment (NMA−P− vs. NMA+P− comparison), including seven molecular functions and one biological process. Interestingly, three groups of six upregulated genes were enriched for response to abscisic acid binding (GO:0010427), hormone binding (GO:0042562), and protein phosphatase inhibitor activity (GO:0004864; Figure 7A). Pathogen infection (NMA−P− vs. NMA−P+ comparison) enriched 5 groups of upregulated 22, 22, 22, 9, and 9 genes for response to abscisic acid binding (GO:0010427), isoprenoid binding (GO:0019840), hormone binding (GO:0042562), chitin metabolic process (GO:0006030), and chitin catabolic process (GO:0006032), respectively (Figure 7B). Aphid and pathogen infection together (NMA − P− vs. NMA + P+ comparison) significantly enriched with 192 GO terms, including 91 biological processes, 97 molecular functions, and four cellular components (*P*_adjust_ < 0.05). The aphid and pathogen together also enriched 11 and 12 upregulated genes for cinnamic acid (GO:0009800) and JA biosynthetic processes (GO:0009695; Figure 7C).

Pathogen infection in AM plant (AMA−P− vs. AMA−P+ comparison) significantly enriched for 126 GO terms, including 55 biological processes, 68 molecular functions, and three cellular components (*P*_adjust_ < 0.05). The enriched GO terms included isoprenoid binding (GO:0019840), hormone binding (GO:0042562), secondary metabolite biosynthetic process (GO:0044550), and oxidoreductase activity, acting on diphenols and related substances as donors, oxygen as acceptor (GO:0016682), and cell wall macromolecule catabolic process (GO:0016998; Figure 7D). Aphid and pathogen infection in AM plant (AMA−P− vs. AMA+P+ comparison) significantly enriched in 121 GO terms, including 50 biological processes, 68 molecular functions, and three cellular components (*P*_adjust_ < 0.05). The AMA−P− vs. AMA+P+ comparison enriched 27, 16, 16, and 12 upregulated genes for isoprenoid binding (GO:0019840), chitin metabolic process (GO:0006030), chitin catabolic process (GO:0006032), and transmitter-gated ion channel activity (GO:0022824), respectively (Figure 7E).

The inoculation of AM fungus with aphid infestation (NMA + P− vs. AMA+P− comparison) enriched in 8 GO terms, including seven biological processes and one cellular component (*P*_adjust_ < 0.05). The 8 GO terms enriched eight, seven, and four DEGs for regulation of jasmonic acid-mediated signaling pathway (GO:2000022), response to blue light (GO:0009637), and organic cation transport (GO:0015695), respectively (Figure 7F). The inoculation of AM fungus with pathogen infection (NMA−P+ vs. AMA−P+ comparison) had nine GO terms significantly enriched DEGs consisted of five molecular functions and four cellular components (*P*_adjust_ < 0.05). The Cdc73/Paf1 complex (GO:0016593), phosphoserine residue binding (GO:0050815), and phosphoprotein binding (GO:0051219) were significantly enriched by six, five, and five downregulated genes, respectively, in the NMA−P+ vs. AMA−P+ comparison (Figure 7G). The DEGs in AM fungus alone (NMA−P− vs. AMA−P−), AM fungus with aphid and pathogen (NMA+P+ vs. AMA+P+) were not significantly enriched for any GO term (Supplementary Table S5).

Aphid infestation in NM (NMA−P− vs. NMA+P− comparison), pathogen infection in NM (NMA−P− vs. NMA−P+ comparison), pathogen in AM (AMA−P− vs. AMA−P+ comparison) enriched 6, 22 and 25 upregulated DEG response to hormone binding (GO:0042562; Supplementary Figure S2A). 22 upregulated and five downregulated DEGs were enriched in the pathogen of AM (AMA−P− vs. AMA−P+) comparison enriched DEGs response to secondary metabolite biosynthetic process (GO:0044550; Supplementary Figure S2B).

### 3.8. KEGG pathway enrichment analysis

AM fungus alone (NMA−P− vs. AMA−P− comparison) and aphid in NM (NMA−P− vs. NMA+P− comparison) were not significantly enriched for any KEGG pathway, whereas the DEGs in the pathogen infected NM plant (NMA−P− vs. NMA−P+ comparison) and pathogen and aphid together infected NM (NMA−P− vs. NMA+P+ comparison) were significantly enriched with 10 and 18 KEGG pathways, respectively (*P*_adjust_ < 0.05).

**Figure 8:**
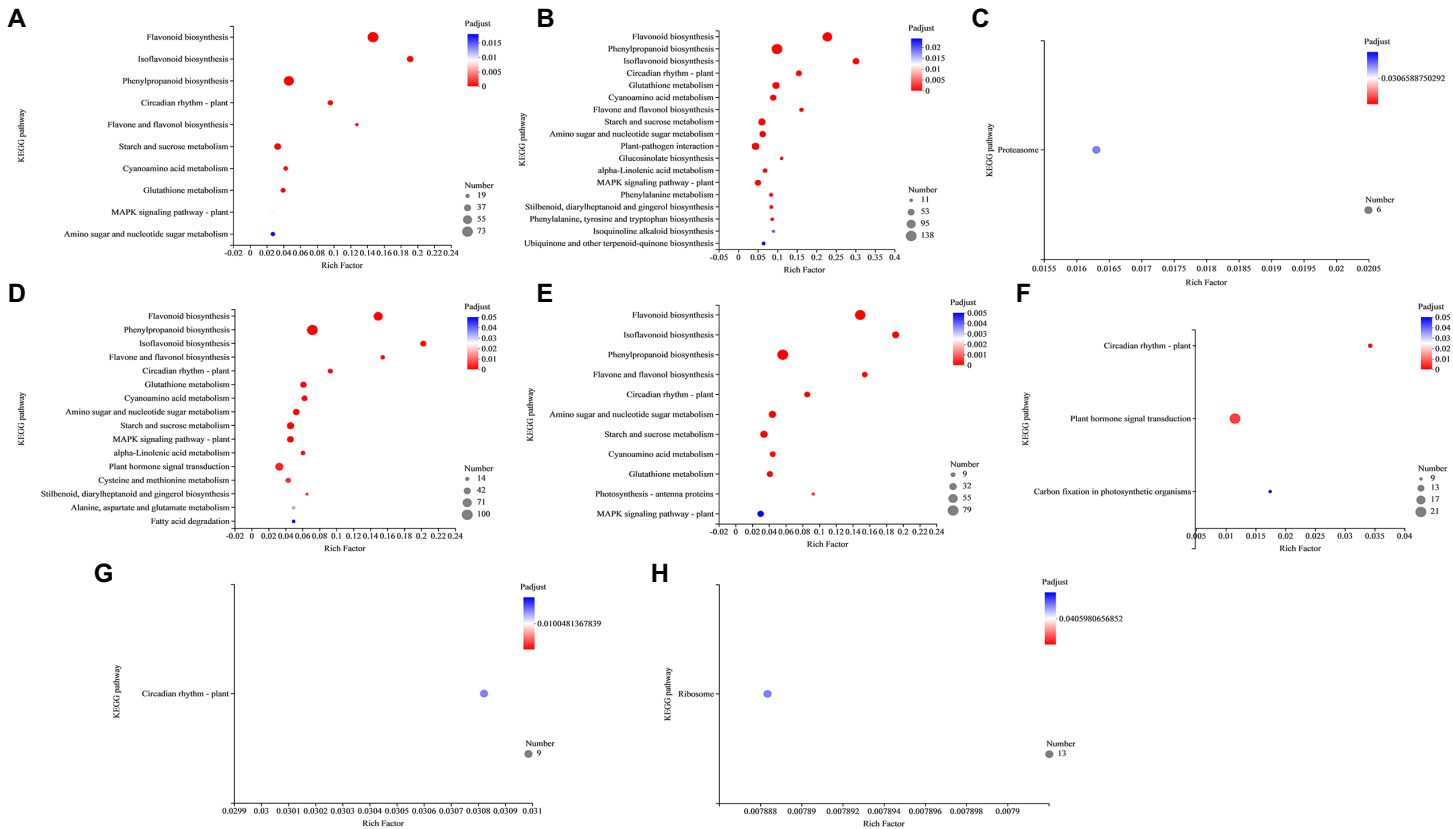
KEGG pathway enrichment analysis in the comparisons **(A)** NMA−P− vs. NMA−P+, **(B)** NMA−P− vs. NMA+P+, **(C)** AMA−P− vs. AMA+P−, **(D)** AMA−P− vs. AMA−P+, **(E)** AMA−P− vs. AMA+P+, **(F)** NMA−P− vs. AMA+P−, **(G)** NMA−P+ vs. AMA−P+, and **(H)** NMA+P+ vs. AMA+P+. The top 20 KEGG pathways are displayed based on differentially expressed genes (*p*_adjust_ < 0.05). The *x*-axis displays the rich factor, which is the ratio of enriched genes to annotated genes; the larger the rich factor, the more strongly significant the enrichment. The *y*-axis shows the KEGG pathway. The point size represents the number of genes and the point color indicates the *P*_adjust_ value. KEGG, Kyoto Encyclopedia of Genes and Genomes.

In the pathogen infected NM plant comparison, 73, 19, 37, 26, and 26 DEGs were enriched in flavonoid biosynthesis (map00941), flavone and flavonol biosynthesis (map00944), starch and sucrose metabolism (map00500), glutathione metabolism (map00480), and MAPK signaling pathway – plant (map04016), respectively (Figure 8A). Moreover, 71, 48, and 17 DEGs from the pathogen and aphid together infected NM comparison were enriched in KEGG pathways for plant–pathogen interaction (map04626), MAPK signaling pathway – plant (map04016), and ubiquinone and other terpenoid-quinone biosynthesis (map00130), respectively (Figure 8B).

Interestingly, one upregulated gene from the aphid infested AM treatment (AMA−P− vs. AMA+P− comparison) was enriched for the genetic information processing pathway (map03050; *P*_adjust_ < 0.05), and 6 genes were enriched for the proteasome pathway (Figure 8C). The 100, 59, and 44 genes that were upregulated in the pathogen infected AM treatments (AMA−P− vs. AMA−P+ comparison; *P*_adjust_ < 0.05) were significantly enriched in phenylpropanoid biosynthesis (map00940), plant hormone signal transduction (map04075), and MAPK signaling pathway – plant (map04016), respectively (Figure 8D). Nine downregulated genes from the aphid and pathogen together infected AM treatment (AMA−P− vs. AMA+P+ comparison) group were enriched in photosynthesis – antenna proteins (map00196). In contrast, 29 genes from the same group were enriched for MAPK signaling pathway – plant (map04016; *P*_adjust_ < 0.05; Figure 8E).

Ten DEGs from the AM fungus and aphid treatment (NMA+P− vs. AMA+P− comparison) were enriched in the circadian rhythm–plant pathways (map04712). An additional 21 and 9 genes (*P*_adjust_ < 0.05) were enriched in pathways for plant hormone signal transduction (map04075) and carbon fixation in photosynthetic organisms (map00710), respectively (Figure 8F). The DEGs from the AM fungus and pathogen treatment (NMA−P+ vs. AMA−P+ comparison) and AM fungus, aphid treatment and pathogen treatment (NMA+P+ vs. AMA+P+ comparison; *P*_adjust_ < 0.05) were significantly enriched only in circadian rhythm – plant (map04712) and ribosome (map03010; Figures 8G,H; Supplementary Table S6).

### 3.9. qRT-PCR validation

Ten genes were randomly selected to validate the RNA-Seq results. Supplementary Table S2 contains the qRT-PCR primer information for the 10 genes. The relative expression levels of seven of the 10 genes were similar to those of the RNA-Seq results (Supplementary Figure S3).

## 4. Discussion

This study established an AM fungus–alfalfa–pea aphid–pathogen interaction system to clarify the effect of an AM fungus on alfalfa response to *P. medicagonis* infection activated by pea aphid infestation. Although pea aphid feeding increased the activity of the plant defense enzyme PPO, aphids increased the severity of leaf spot disease caused by *P. medicaginis*. Sucking insects, such as aphids, damage plants directly and function as pathogen vectors (Hendry et al., 2018). Aphids pierce the leaves and stems of the host-plant phloem cells and extract the phloem sap using stylets (Cook et al., 2015). Pea aphids cause wounds on alfalfa, which provide potential entry points for pathogen invasion. As expected, the AM fungus enhanced nitrogen and phosphorus uptake by the plant, promoted plant growth, increased the alfalfa JA and ABA contents in response to aphid attack and pathogen infection, and reduced the disease severity of alfalfa infested or not infested with aphids. In addition, the KEGG ribosome pathway was significantly enriched between mycorrhizal and non-mycorrhizal alfalfa infested by aphids or infected by *P. medicaginis* alone and the combination of aphid and pathogen, which indicated that the AM fungus *R. intraradices* may affect the plant responses to insect and pathogen attack by regulating the ribosome pathway.

Previous studies have shown that biotic factors either increase (Brito et al., 2019), have no influence (Li et al., 2019), or decrease (Khan et al., 2010) AM colonization. This difference is related with the combination of plant, AM fungi and the biotic stresses. E.g., Mycorrhizal colonization of 8 preceding crop was significantly suppressed by *Fusarium oxysporum* inoculation with field soil contained *Glomus mosseae* and *G. fasciculatum* (Khan et al., 2010). While aphid infestation does not affect AM colonization of alfalfa inoculated with *R. intraradices* (Li et al., 2019). Interestingly, neither pea aphid infestation nor *P. medicaginis* infection affected colonization by the AM fungus (~45%). This observation is in agreement with previous findings that an AM fungus (*Claroideoglomus etunicatum*) remains stable in its colonization of perennial ryegrass (*Lolium perenne*) under simultaneous stress from the fungal pathogen *Bipolaris sorokiniana* and a grass endophyte (*Epichloë*; Li et al., 2018).

Infestation by pea aphids and infection by *P. medicaginis* stimulated increased enzyme activities in the host plant as a defense response. Aphid infestation induced PPO activity, whereas pathogen infection increased the activities of CAT and POD in the plant. Each of these enzymes is important for the defense of the host plant to aphids and *P. medicaginis* (Li et al., 2019, Li Y et al., 2021). The GO analysis also shows that infestation of pea aphid and pathogen enriched different numbers of GO terms response for DEGs including plant defence and signal materials such as chitinase and ABA. ABA is an important plant hormone in abiotic stress, exerts a positive role in activating the plants’ own defense mechanisms (Liu et al., 2017). And chitinase is key player in the plant immune system that offers successful protection to fungal infections over a broad spectrum of diseases (Mir et al., 2021). Aphid-infested plants developed severe spring black stem and leaf spot disease, indicating that the positive effects of the aphid-induced defense enzyme PPO were overridden by the adverse effects, which included the provision of wounds that enabled pathogen infection. The severe disease incidence in aphid treatment and the higher value of GO terms (192) response for more DEGs in the combination of aphid and pathogen treatment also indicates plant was under severe stress and more of plant defence related materials were activated when exposed to the combination of aphid and pathogen (Li Y.D. et al., 2021).

AM fungus mostly benefit host plant under biotic and abiotic stress (Smith and Read, 2008). The AM fungus enhanced the JA content and improved plant resistance against subsequent *P. medicaginis* infection, as shown by the lower disease severity in mycorrhizal alfalfa. The AM fungus also increased the ABA content in plants exposed to pathogen infection, possibly enhancing the resistance of the plant to the simultaneous stresses of aphid infestation and pathogen infection (Li et al., 2019, Li Y. et al., 2021). The inoculation of AM fungus enriched 8 GO terms with three upregulated DEGs of JA-mediated signaling pathway (GO:2000022). Thus, our first hypothesis that plant defense profiles will differ with AM colonization, aphid infestation and pathogen infection was supported. Both JA and ABA are hormones important for induction of systemic disease resistance (Yang et al., 2015). After successful establishment of a pathogen within plant tissues, ABA acts synergistically with other hormonal pathways to enhance plant defense (Anderson et al., 2005). Therefore, ABA cooperates with JA as a strong modulator to induce JA-mediated defense (Proietti et al., 2018). Our second hypothesis, that an AM fungus will enhance pea aphid-induced plant defense response against subsequent pathogen infection, was also upheld from the perspective of plant defense enzyme activities and JA, even for an aphid-infested plant that showed higher disease severity.

Salicylic acid is important for plant resistance to aphid infestation and pathogen infection. The results of the present transcriptomic analysis were consistent with the significant increase in SA content in plants either infested with pea aphids or infected by *P. medicaginis*, and in plants under dual attack from aphids and *P. medicaginis*. The SA signals result in acquisition of plant resistance to aphids and pathogens (Chaman et al., 2003), and are crucial signaling components for active plant defense (Durner et al., 1997). Previous research involving the SA-metabolizing *NahG* transgene expressed in the *Mi-1* background demonstrated that SA regulates resistance to aphids (Li et al., 2006). Moreover, early and strong induction of specific genes responsive to SA may initiate resistance against aphid infestation in *M. truncatula* (Gao et al., 2008). Accumulation of SA and pathogenesis-related (PR) gene expression can induce systemic acquired resistance (Dempsey and Klessig, 2012; Fu and Dong, 2013). In the present study, the increase in SA content induced by aphid infestation remained stable before and after pathogen infection. Therefore, we assume that the enhanced accumulation of SA improved the plant resistance to subsequent pathogen infection.

The GO terms analysis shows AM fungus changed plant responses to pea aphid and pathogen infection. Mycorrhizal alfalfa enriched more GO terms than non-mycorrhizal alfalfa under pathogen infestation. The enriched GO terms and KEGG pathway of pea aphid and pathogen were completely different in AM plant and NM plants. Colonization by the AM fungus affected hormone binding (GO:0042562), secondary metabolite biosynthetic process (GO:0044550), and isoprenoid binding (GO:0019840) in alfalfa, thus influencing the plant response to aphid infestation and pathogen infection.

The RNA-Seq results showed that genes associated with the GO terms abscisic acid binding (GO:0010427) and hormone binding (GO:0042562) were upregulated in alfalfa plants infested with aphids or infected by *P. medicaginis*. These results were consistent with the higher contents of ABA and SA, and increased activities of plant defense enzymes, such as CAT and PPO, in alfalfa plants either infested with aphids or infected by *P. medicaginis*, and in plants exposed to both aphid infestation and pathogen infection.

The RNA-Seq results also revealed that genes associated with the mitogen-activated protein kinase (MAPK) signaling pathway – plant (map04016) were upregulated in alfalfa infested with aphids and infected with *P. medicaginis*. The MAPK cascades play a role in activating multiple aspects of the plant immune system in response to pathogen infection (Zipfel et al., 2006; Miya et al., 2007). In alfalfa, genes associated with the chitin metabolic process (GO:0006030) and chitin catabolic process (GO:0006032) were upregulated in response to *P. medicaginis* infection. Chitinases are typically enhanced in plants challenged by aphids (Ye et al., 2019) and fungal pathogens (Mauch et al., 1988). Chitinase hydrolyze fungal cell walls that are composed of chitin, thus preventing fungal growth and aphid feeding (Theis and Stahl, 2004; Rajendran et al., 2011). Therefore, the induction of genes associated with the chitin metabolic process by aphids may also improve plant resistance to pathogens.

In conclusion, in the alfalfa–AM fungus–insect–pathogen system, aphid infestation led to severe disease incidence resulting from *P. medicaginis* infection. Enzymes associated with plant defense and the MAPK signaling pathway induced by aphid infestation did not offset the negative impact of aphid feeding on the diseased portions of the plants. The AM fungus altered the response of plants to pathogen infection facilitated by pea aphid infestation through promoting the uptake of nitrogen and phosphorus by the plant, enhancing the activities of defense-related enzymes and signaling substances, such as JA and ABA, as well as the expression of genes associated with plant defense and physiological and chemical metabolic pathways. This study affords knowledge of AM fungus-mediated plant responses to insect infestation and the subsequent pathogen infection.

## Data availability statement

The datasets presented in this study can be found in online repositories. The names of the repository/repositories and accession number(s) can be found in the article/Supplementary material.

## Author contributions

TD and YW: conceptualization and experimental design. YW and YL: investigation. YW: formal analysis. YW, YL, and TD: writing–original draft, review and editing. All authors contributed to the article and approved the submitted version.

## Funding

This research was supported by the National Natural Science Foundation of China (3207141294), the China Modern Agriculture Research System (CARS-22 Green Manure).

## Conflict of interest

The authors declare that the research was conducted in the absence of any commercial or financial relationships that could be construed as a potential conflict of interest.

## Publisher’s note

All claims expressed in this article are solely those of the authors and do not necessarily represent those of their affiliated organizations, or those of the publisher, the editors and the reviewers. Any product that may be evaluated in this article, or claim that may be made by its manufacturer, is not guaranteed or endorsed by the publisher.

## Footnotes

^1^ https://github.com/jstjohn/SeqPrep

^2^ https://github.com/najoshi/sickle

^3^ https://figshare.com/projects/whole_genome_sequencing_and_assembly_of_Medicago_sativa/66380

^4^ http://deweylab.biostat.wisc.edu/rsem/

^5^ https://github.com/tanghaibao/Goatools

^6^ www.majorbio.com

## Supplementary material

SUPPLEMENTARY FIGURE S1 The measurement of samples of the study.

SUPPLEMENTARY FIGURE S2 Heatmap analysis of the **(A)** Homone binding, **(B)** Secondary metabolite biosynthetic process in the NMA−P−, NMA+P−, NMA−P+, NMA+P+, AMA−P−, AMA+P−, AMA−P+ and AMA−P+.

SUPPLEMENTARY FIGURE S3 Gene expression levels obtained from RNA-Seq (blue) and qRT-PCR data (red) of seven genes in all treatments. The left *y*-axis shows the qRT-PCR data (2^−∆∆*C*q^). The right *y*-axis shows the RNA-Seq data (FPKM). FPKM, fragments per kilobase of exon per million mapped reads; qRT-PCR, real-time quantitative reverse-transcription PCR.
