# Supplementary figures and images for: Arbuscular mycorrhizal fungus changes alfalfa response to pathogen infection activated by pea aphid infestation
Source: Front Microbiol. 2023 Feb 8;13:1074592. doi: 10.3389/fmicb.2022.1074592 (PMC9945236; doi:10.3389/fmicb.2022.1074592)

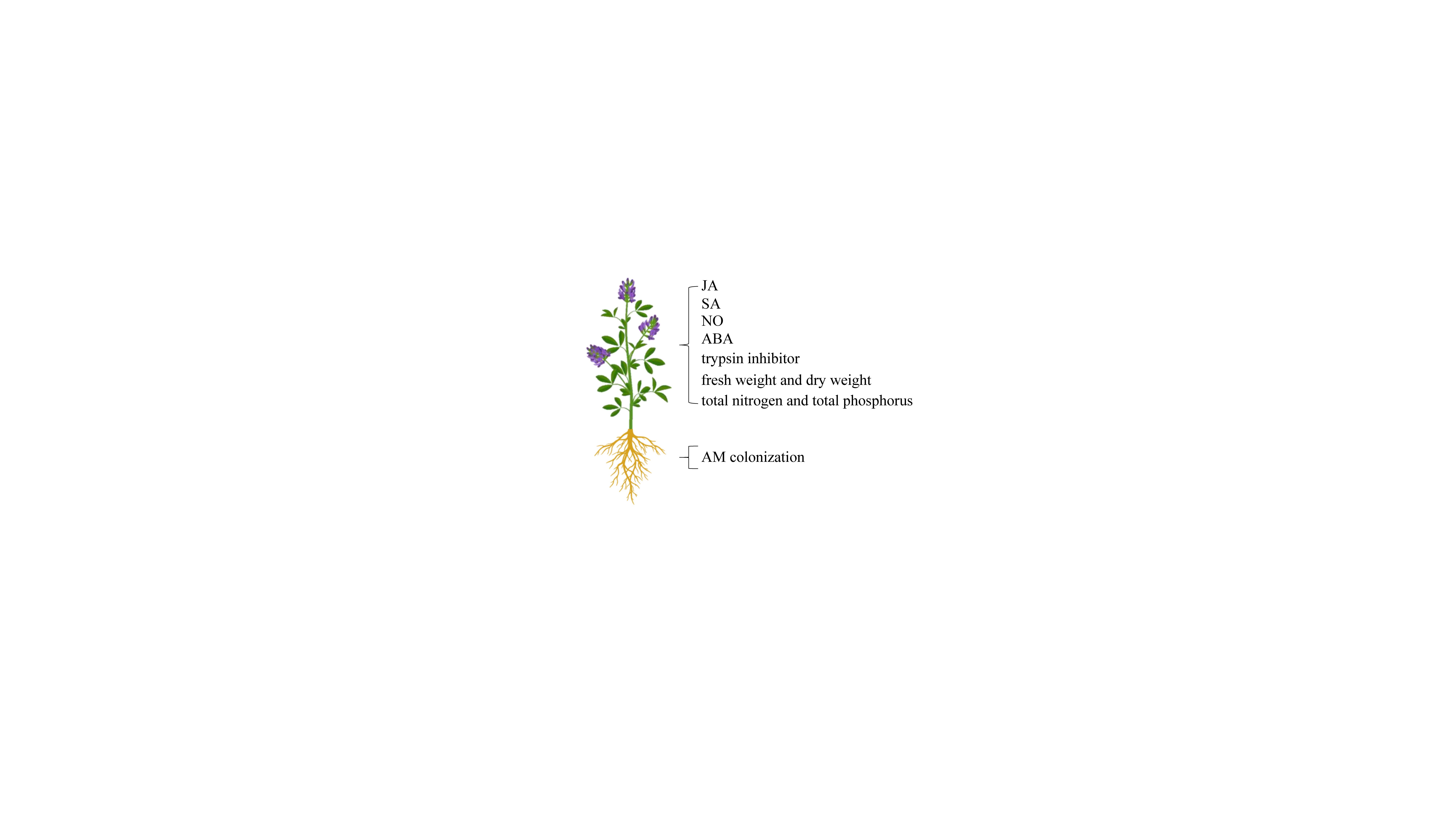

Supplement: SUPPLEMENTARY FIGURE S1 — The measurement of samples of the study. [file Image_1.TIF]
