# Supplementary material for: Arbuscular mycorrhizal fungus changes alfalfa response to pathogen infection activated by pea aphid infestation
Source: Front Microbiol. 2023 Feb 8;13:1074592. doi: 10.3389/fmicb.2022.1074592 (PMC9945236; doi:10.3389/fmicb.2022.1074592)
Supplement: Supplementary file 5 [file Table_2.DOCX]

Supplementary table 2． Gene primer information was verified by q RT-PCR

| Gene ID | primer (5’-3’) | primer (3’-5’) |
| --- | --- | --- |
| MS.gene041629 | ACTGAAGGAACTGGTGGTGC | TCCAGTGCCCCCAATAATGC |
| MS.gene055964 | GACGTTATGGAAGGCCCCAA | TTCGAGTCAAGTTCAGGCCC |
| MS.gene52002 | GCCAAGGCTTTGAGGCTTTC | GTAGCCTCTTCAGCCGGTTT |
| MS.gene32028 | CTCTCCCACCTTCGAGGAGA | TCTCTGTCGGCCGGATCTAA |
| MS.gene051780 | ATCCCCCAACAACCTCCCTC | CCATGGAGCGGATTCAGCAT |
| MS.gene001519 | AAAGCAGTCAGGATGGTGGG | TGGTCATGCCATCCATTGCT |
| MS.gene00217 | GGCTCTTGAAGGTTACGTTTTGG | AGGGAGGTACACATATGACTTACAA |
| MS.gene059052 | ACATGTCTCAACCGTGGCTC | TTCAACACTTTGGGCAGCAG |
| MS.gene00225 | CAAAGGTGATTTCTGCTGCCC | AGCACAAAGTTGGTTTTGCCA |
| MS.gene059046 | AGGTGATTTCTGCTGCCCAA | CCCTGTTCCTCCCACTAAGC |
