# Supplementary material for: Arbuscular mycorrhizal fungus changes alfalfa response to pathogen infection activated by pea aphid infestation
Source: Front Microbiol. 2023 Feb 8;13:1074592. doi: 10.3389/fmicb.2022.1074592 (PMC9945236; doi:10.3389/fmicb.2022.1074592)
Supplement: Supplementary file 6 [file Table_3.DOCX]

Supplementary table 3. Main effects of AM fungus (inoculation or uninculation), pea aphid (infested or uninfested), pathogen (infected or uninfected), and their two way and three way interactions on AM colonization, Shoot fresh weight, Shoot dry weight, Shoot total N, Shoot total P, Disease incidence, Disease index, PPO, CAT, POD, SOD, SA, JA, ABA, NO, Trypsin inhibitor, Total phenols. *P*＜0.05 are highlighted in bold.

|  | AM fungus | | | Aphid | | | Pathogen | | | AM fungus*Aphid | | | AM fungus*Pathogen | | | Aphid*Pathogen | | | AM fungus*Aphid*Pathogen | | |
| --- | --- | --- | --- | --- | --- | --- | --- | --- | --- | --- | --- | --- | --- | --- | --- | --- | --- | --- | --- | --- | --- |
|  | DF | *F* | *P* | DF | *F* | *P* | DF | *F* | *P* | DF | *F* | *P* | DF | *F* | *P* | DF | *F* | *P* | DF | *F* | *P* |
| AM colonization | 1 | 1215 | **<.0001** | 1 | 0.1000 | 0.5331 | 1 | 0.0402 | 0.2180 | 3 | 389.0667 | 0.5331 | 3 | 426.4167 | 0.2180 | 3 | 0.0188 | 0.5331 | 7 | 167.3143 | 0.5331 |
| Shoot fresh weight | 1 | 5.4717 | **0.0040** | 1 | 0.8172 | 0.7334 | 1 | 18.4966 | **<.0001** | 3 | 1.7567 | 0.7500 | 3 | 14.0400 | **0.0498** | 3 | 6.5729 | 0.1732 | 7 | 5.9467 | 0.9094 |
| Shoot dry weight | 1 | 8.7180 | **0.0102** | 1 | 0.4718 | 0.4715 | 1 | 0.5727 | 0.4289 | 3 | 2.9947 | 0.7819 | 3 | 3.7091 | 0.2484 | 3 | 0.3665 | 0.7287 | 7 | 1.5096 | 0.9925 |
| Shoot total N | 1 | 4.9955 | **0.0475** | 1 | 1.1206 | 0.2882 | 1 | 0.0384 | 0.8377 | 3 | 2.4712 | 0.3519 | 3 | 2.1524 | 0.2498 | 3 | 0.4957 | 0.5014 | 7 | 1.2715 | 0.6424 |
| Shoot total P | 1 | 3.7239 | **0.0459** | 1 | 2.3527 | 0.0961 | 1 | 0.1595 | 0.6359 | 3 | 5.9642 | **0.0147** | 3 | 1.1951 | 0.9689 | 3 | 0.8347 | 0.6298 | 7 | 2.3370 | 0.4486 |
| Disease incidence | 1 | 3.4726 | **0.0294** | 1 | 7.1985 | **0.0097** | - | - | - | 3 | 6.3911 | 0.3984 | - | - | - | - | - | - | - | - | - |
| Disease index | 1 | 11.2493 | **0.0020** | 1 | 3.1425 | **0.0166** | - | - | - | 3 | 10.0424 | 0.3899 | - | - | - | - | - | - | - | - | - |
| PPO | 1 | 1.0739 | 0.1950 | 1 | 12.4401 | **0.0017** | 1 | 3.0242 | **0.0446** | 3 | 5.0383 | 0.3589 | 3 | 1.3404 | 0.9884 | 3 | 7.3230 | 0.2202 | 7 | 3.3311 | 0.9061 |
| CAT | 1 | 0.0136 | 0.8979 | 1 | 0.0307 | 0.8473 | 1 | 14.2212 | **0.0047** | 3 | 0.0946 | 0.5742 | 3 | 4.3649 | 0.7992 | 3 | 4.3559 | 0.8603 | 7 | 1.6376 | 0.6626 |
| POD | 1 | 0.4275 | 0.5050 | 1 | 1.5143 | 0.2280 | 1 | 5.5041 | **0.0420** | 3 | 0.8438 | 0.4135 | 3 | 2.0690 | 0.5208 | 3 | 2.4545 | 0.7426 | 7 | 1.2003 | 0.6348 |
| SOD | 1 | 1.7220 | 0.2129 | 1 | 0.0170 | 0.8952 | 1 | 4.8255 | 0.0580 | 3 | 0.5311 | 0.9229 | 3 | 2.5295 | 0.4797 | 3 | 1.7103 | 0.4710 | 7 | 1.0268 | 0.6330 |
| SA | 1 | 1.3234 | 0.1090 | 1 | 1.1370 | 0.1349 | 1 | 12.4969 | **0.0002** | 3 | 1.3058 | 0.1024 | 3 | 5.8813 | 0.0820 | 3 | 8.8633 | **0.0038** | 7 | 5.9391 | 0.4158 |
| JA | 1 | 5.0587 | **0.0259** | 1 | 3.4653 | 0.0556 | 1 | 0.7850 | 0.3280 | 3 | 3.0850 | 0.8810 | 3 | 3.6235 | **0.0491** | 3 | 1.3887 | 0.8928 | 7 | 2.1569 | 0.7877 |
| ABA | 1 | 0.0218 | 0.8747 | 1 | 0.7734 | 0.3573 | 1 | 0.2274 | 0.6123 | 3 | 0.4638 | 0.3942 | 3 | 3.3439 | **0.0063** | 3 | 0.3159 | 0.9581 | 7 | 1.5781 | 0.6874 |
| NO | 1 | 2.0884 | 0.0621 | 1 | 0.0103 | 0.8880 | 1 | 23.5678 | **<.0001** | 3 | 0.6592 | 0.8603 | 3 | 10.7865 | 0.1890 | 3 | 8.9629 | 0.1006 | 7 | 4.9763 | 0.5745 |
| Trypsin inhibitor | 1 | 0.5295 | 0.4294 | 1 | 0.6650 | 0.3812 | 1 | 10.5951 | **0.0047** | 3 | 0.6242 | 0.3540 | 3 | 3.9785 | 0.3905 | 3 | 3.7303 | 0.7335 | 7 | 1.8929 | 0.5830 |
| Total phenols | 1 | 2.1207 | 0.1721 | 1 | 1.1520 | 0.3033 | 1 | 0.0055 | 0.9416 | 3 | 1.4830 | 0.3237 | 3 | 0.5694 | 0.8146 | 3 | 0.5642 | 0.4485 | 7 | 0.8470 | 0.2901 |
