# Supplementary material for: Arbuscular mycorrhizal fungus changes alfalfa response to pathogen infection activated by pea aphid infestation
Source: Front Microbiol. 2023 Feb 8;13:1074592. doi: 10.3389/fmicb.2022.1074592 (PMC9945236; doi:10.3389/fmicb.2022.1074592)
Supplement: Supplementary file 7 [file Table_4.DOCX]

Supplementary table 4． Summary statistics for sequencing for four libraries from NMA-P-, NMA+P-, NMA-P+, NMA+P+, AMA-P-, AMA+P-, AMA-P+ and AMA+P+ samples and three replicates per sample.

| Sample name | Raw reads | Clean reads | GC content(%) | Q20(%) | Q30(%) | Uniquely mapped(%) | Multiple mapped(%) | Unmapped(%) |
| --- | --- | --- | --- | --- | --- | --- | --- | --- |
| NMA-P-1 | 52,304,792 | 51,810,754 | 43.08 | 97.88 | 93.82 | 40.09 | 51.84 | 8.07 |
| NMA-P-2 | 46,473,094 | 46,147,370 | 42.98 | 98.24 | 94.66 | 40.98 | 51.67 | 7.35 |
| NMA-P-3 | 48,468,488 | 48,075,260 | 42.83 | 98.20 | 94.56 | 41.52 | 51.05 | 7.43 |
| NMA+P-1 | 53,568,978 | 53,208,740 | 43.70 | 98.35 | 94.92 | 37.95 | 47.51 | 14.54 |
| NMA+P-2 | 42,919,558 | 42,437,864 | 41.97 | 98.25 | 94.70 | 41.16 | 50.95 | 7.89 |
| NMA+P-3 | 41,839,006 | 41,156,196 | 42.00 | 98.18 | 94.59 | 36.54 | 45.50 | 17.96 |
| NMA-P+1 | 46,625,196 | 46,240,394 | 43.18 | 98.15 | 94.45 | 38.66 | 49.38 | 11.96 |
| NMA-P+2 | 48,389,898 | 47,960,396 | 42.96 | 98.02 | 94.13 | 39.05 | 48.77 | 12.18 |
| NMA-P+3 | 53,394,368 | 52,999,082 | 42.91 | 98.18 | 94.50 | 35.09 | 44.78 | 20.13 |
| NMA+P+1 | 47,010,342 | 46,620,236 | 42.93 | 98.17 | 94.49 | 39.65 | 48.77 | 11.58 |
| NMA+P+2 | 45,503,264 | 45,017,358 | 41.95 | 98.14 | 94.44 | 37.70 | 44.17 | 18.13 |
| NMA+P+3 | 49,320,988 | 48,953,192 | 42.33 | 98.07 | 94.25 | 40.55 | 50.29 | 9.16 |
| AMA-P-1 | 41,604,716 | 40,936,666 | 42.55 | 98.03 | 94.22 | 41.66 | 47.48 | 10.86 |
| AMA-P-2 | 49,398,436 | 49,007,946 | 43.14 | 98.21 | 94.56 | 41.72 | 50.84 | 7.44 |
| AMA-P-3 | 46,206,932 | 45,666,380 | 44.28 | 98.24 | 94.73 | 35.40 | 42.60 | 22.00 |
| AMA+P-1 | 42,068,260 | 41,430,014 | 42.23 | 98.13 | 94.46 | 41.78 | 49.22 | 9.00 |
| AMA+P-2 | 41,649,346 | 40,986,240 | 42.74 | 98.12 | 94.42 | 38.76 | 45.04 | 16.20 |
| AMA+P-3 | 51,358,066 | 50,925,424 | 43.11 | 98.14 | 94.43 | 39.00 | 48.88 | 12.12 |
| AMA-P+1 | 58,300,788 | 57,771,548 | 44.30 | 98.32 | 94.89 | 35.85 | 45.44 | 18.71 |
| AMA-P+2 | 47,359,286 | 46,491,720 | 49.91 | 98.12 | 94.56 | 21.20 | 21.83 | 56.97 |
| AMA-P+3 | 43,386,308 | 42,786,596 | 48.04 | 98.02 | 94.26 | 24.35 | 26.05 | 49.60 |
| AMA+P+1 | 42,599,980 | 42,127,358 | 43.11 | 98.17 | 94.55 | 35.23 | 41.17 | 23.60 |
| AMA+P+2 | 47,568,912 | 47,220,414 | 43.89 | 98.3 | 94.83 | 34.40 | 42.58 | 23.02 |
| AMA+P+3 | 48,099,958 | 47,743,372 | 43.36 | 98.19 | 94.55 | 38.98 | 46.97 | 14.05 |

Note: AM= inoculated with *R. intraradices*, NM=un-inoculated with *R. intraradices*;

A+= infected by *A. pisum*, A-= un-infested by *A. pisum*;

P+=infected by *P. medicaginis*, P-=un-infested by *P. medicaginis*.
